# Cone beam computed tomography based image guidance and quality assessment of prostate cancer for magnetic resonance imaging-only radiotherapy in the pelvis

**DOI:** 10.1016/j.phro.2021.05.001

**Published:** 2021-05-13

**Authors:** Jens M. Edmund, Daniel Andreasen, Koen Van Leemput

**Affiliations:** a Radiotherapy Research Unit, Department of Oncology, Gentofte and Herlev Hospital, University of Copenhagen, 2730 Herlev, Denmark; b Niels Bohr Institute, University of Copenhagen, 2100 Copenhagen, Denmark; c Department of Health Technology, Technical University of Denmark, 2800 Lyngby, Denmark; d Martinos Center for Biomedical Imaging, Department of Radiology, Massachusetts General Hospital, Harvard Medical School, Boston, MA 02114, USA

**Keywords:** MRI-only RT, IGRT, Synthetic CT, Pseudo CT, Magnetic resonance imaging, Radiotherapy, Cone beam CT

## Abstract

**Background and purpose:**

Radiotherapy (RT) based on magentic resonance imaging (MRI) only is currently used clinically in the pelvis. A synthetic computed tomography (sCT) is needed for dose planning. Here, we investigate the accuracy of cone beam CT (CBCT) based MRI-only image guided RT (IGRT) and sCT image quality.

**Materials and methods:**

CT, MRI and CBCT scans of ten prostate cancer patients were included. The MRI was converted to a sCT using a multi-atlas approach. The sCT, CT and MR images were auto-matched with the CBCT on the bony anatomy. Paired sCT-CT and sCT-CBCT data were created. CT numbers were converted to relative electron (RED) and mass densities (DES) using a standard calibration curve for the CT and sCT. For the CBCT RED/DES conversion, a phantom and paired CT-CBCT population based calibration curve was used. For the latter, the CBCT numbers were averaged in 100 HU bins and the known RED/DES of the CT were assigned. The paired sCT-CT and sCT-CBCT data were averaged in bins of 10 HU or 0.01 RED/DES. The median absolute error (MeAE) between the sCT-CT and sCT-CBCT bins was calculated. Wilcoxon rank-sum tests were carried out for the IGRT and MeAE study.

**Results:**

The mean sCT or MR IGRT difference from CT was ≤ 2 mm but significant differences were observed. A CBCT HU or phantom-based RED/DES MeAE did not estimate the sCT quality similar to a CT based MeAE but the CBCT population-based RED/DES MeAE did.

**Conclusions:**

MRI-only CBCT-based IGRT seems feasible but caution is advised. A MeAE around 0.1 DES could call for sCT quality inspection.

## Highlights

• MRI-only IGRT accuracy is ≤2 mm as compared to CT but significant differences were observed.
• MRI-only CBCT-based IGRT seems feasible but caution is advised.
• The median absolute error (MeAE) for independent verification on the sCT quality is proposed.
• A MeAE around 0.1 in mass density could call for sCT quality inspection.

## 1 Introduction

Radiotherapy (RT) planned on magnetic resonance imaging (MRI) only, commonly referred to as MRI-only RT, is currently implemented clinically for the pelvic region mainly focusing on prostate cancer [1], [2], [3], [4]. A major focus and active area of research within MRI-only RT is the development of methods that convert the MRI into a synthetic computed tomography (sCT) needed for dose planning and possible image guidance (IGRT) purposes [5], [6]. Following the trends in related areas such as computer vision and medical imaging, much attention has recently been given to deep learning convolution neural network techniques [7], [8], [9], [10] and commercial solutions are currently available for clinical use [11], [12].

The vast majority of the MRI-only RT literature has focused on methods for generating the sCT and the corresponding dose planning performance as compared to the CT-based clinical standard. Literature on MRI-only IGRT and independent verification on the sCT quality in the absence of the CT, however, is much more sparse. As a consequence, no clinical guidelines on markerless cone beam CT (CBCT) IGRT and routine sCT quality checks exist for MRI-only RT.

In MRI-only RT, the CBCT attracts attention for sCT assessment since it is the only independent measurement of a CT-like image available. For sCT quality verification, Palmer et al. used the CBCT of the first fraction to assess the dosimetric accuracy of the sCT comparing identical dose calculations based on sCT, CT and CBCT images [13]. sCT-CT and sCT-CBCT dose differences were found to be ≤ 1%.

For MRI-only IGRT, Kemppainen et al. compared sCT-CBCT and CT-CBCT based registrations for different pelvic cancers and found the difference to be ≤ 0.5 mm [14]. One CBCT from a randomly selected fraction was used for 10 patients and both bone and soft-tissue based registrations were included in the study. A similar agreement of ≤ 1 mm and 1° was obtained for 5 prostate patients based on 10 CBCTs of each patient registering the sCT to the CBCT on the volume around the prostate only [15].

In a previous study for the brain, we investigated whether the CBCT could reliably be used to assess the sCT quality and IGRT accuracy in MRI-only RT [16]. The study investigated MRI-CBCT, sCT-CBCT and CT-CBCT differences and demonstrated a promising potential to assess the agreement with a corresponding CT-based RT. Given the current clinical implementation of MRI-only RT in the pelvis, one goal of this study was to examine the general applicability of the previous CBCT-based method for this more challenging anatomical region. In line with [15], we further included more CBCTs of some patients for the IGRT investigation to address the accuracy of CBCT based MRI-only IGRT. Given the fact that MRI-only RT has been adapted into clinical practice, our aims were 1) to provide data for the establishment of an overall agreement of markerless IGRT in the pelvis for MRI-only RT as compared to a standard CT-based workflow, and, 2) provide novel suggestions for an implementable feasible quantitative assessment of sCT image quality.

## 2 Materials and methods

### 2.1 Imaging and pre-processing

The CT and MRI scans of ten prostate cancer RT patients were retrospectively included in the study. Patients informed consent for using their data was obtained. The CT scans were acquired using a standard protocol for pelvic scans (Brilliance Big Bore, Philips Medical Systems, Cleveland, OH, 120 kVp, 232–503 mAs). The voxel resolution was between 0.78 × 0.78 × 2.00 and 1.4 × 1.4 × 2.0 mm for an in‐plane matrix of 512 × 512 voxels and 129–199 slices. The MRI scans were obtained with a *T*_1_‐weighted sequence, TE/TR = 10/623 ms, on a 1 T open scanner (Panorama HFO, Philips Medical Systems, Cleveland, OH) using a bridge body coil. The voxel resolution was 0.8 × 0.8 × 4.0 mm, for an in‐plane matrix between 528 × 528 and 640 × 640 voxels and 16–24 slices. The MRI has a large field of view (FOV) to include the outer body contour needed for sCT generation. The patients were positioned in treatment position using the same fixation devices during both the MRI and CT scanning. In addition, a CBCT scan was obtained for each patient during the course of RT. The CBCTs were acquired with the On-Board Imager (OBI) system mounted on the linac (models iX and TrueBeam, Varian Medical Systems Inc., Palo Alto, CA, USA) using an abdominal scanning protocol of 125 kV and mAs 659–1049 with a resolution of 0.9 × 0.9 × 2.0 mm or 1.2 × 1.2 × 2.0–2.5 for an in-plane matrix of 512 × 512 or 384 × 384 voxels, respectively. Eight and nine weekly CBCTs were further included for patient 7 and 9,^1^ respectively.

The MRI was deformably (non-linearly) aligned with the corresponding CT using elastix software and checked by visually inspecting [17]. The MRI was then re-sampled to match the CT resolution. A sCT was generated from each patient’s MRI using a patch-based approach trained on the non‐linearly co‐registered MRI and CT multi-atlas of the other nine patients [18], [19], [20]. For each MRI voxel, a 3D subvolume of voxels (a patch) was extracted and the most similar patches in the multi-atlas were found using the *L*_2_-normalized intensity distance. A weighted average of the corresponding CT values in the multi-atlas was then assigning to the MRI voxel. The sCT resolution was identical to that of the MRI, i.e. the resolution of the CT. Full details of the sCT method can be found in [21]. For the sCT quality assessment, the CBCT was rigidly aligned with and re-sampled to the resolution of the CT. For the IGRT study, the CBCT maintained its original resolution.

### 2.2 IGRT study

The CT, MRI and sCT scans were aligned with each other given the pre-processing procedure. The scans were imported into the registration workspace in Eclipse v.15.6 (Varian Medical Systems, Helsinki, Finland). Here, the scans were roughly aligned manually with the CBCT followed by an auto-match on the bony anatomy in line with the clinical matching procedure for elective lymph node irradiation. As the sCT did not contain prostate markers, this markerless match strategy was chosen. Both translational (AP: anterior-posterior, LR: left–right, CC: cranio-caudal) and rotational (pitch, roll, jaw) displacements, e.g. 6 degrees of freedom (6 DOF), were included in the matches. For the CT and sCT reference, the bone anatomy included voxels between 100 and 4000 hounsfield unit (HU) while no intensity constraints were applied on the MRI-CBCT matches. All matches were visually inspected for acceptable agreement. The MRI-CBCT (ΔMRI) and sCT-CBCT (ΔsCT) difference relative to the CT-CBCT registration was calculated for each DOF and pooled for one CBCT from all 10 patients. The same procedure was done on the weekly CBCTs of patient 7 and 9, respectively. A Shapiro-Wilk test showed that ΔMRI and ΔsCT were not normally distributed (p < 0.02 for all tests). A paired Wilcoxon rank-sum test was consequently performed to determine significant difference from 0 (the CT-CBCT reference) [22].

### 2.3 sCT quality assessment

**Fig. 1:**
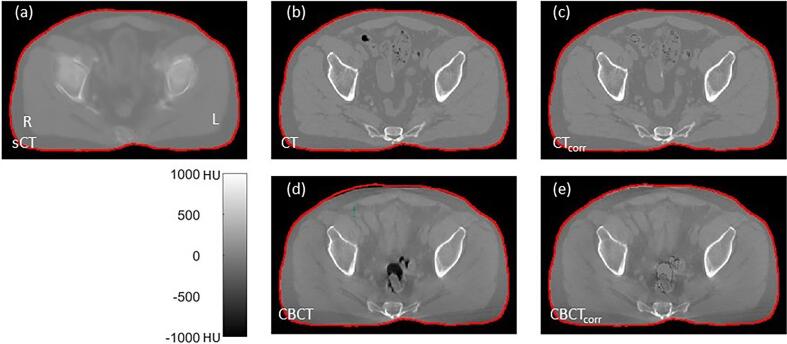
Correction strategy for synthetic CT quality assessment. (a) Synthetic CT = sCT. Patient orientation indicated by right = R and left = L. (b) Computed tomography = CT. (c) Corrected CT = CT_corr_ with air cavities and body outline difference as compared to the sCT filled with water. (d) Cone beam CT = CBCT. (d) Corrected CBCT = CBCT_corr_. The red contour is the body outline of the sCT. Grayscale is in Hounsfield units (HU). The CT voxel resolution was between 0.78 × 0.78 × 2.00 and 1.4 × 1.4 × 2.0 mm for an in‐plane matrix of 512 × 512 voxels. The sCT and CBCT were re-sampled to the resolution of the CT. (For interpretation of the references to colour in this figure legend, the reader is referred to the web version of this article.)

Paired sCT-CT and sCT-CBCT data were created and cropped to the (smallest) body outline of sCT. To compensate for temporary deformations in air pockets and body outline between the sCT, CT and CBCT, which are not relevant for sCT quality assessment and hence should be eliminated or reduced to a minimum, water was assigned to the CBCT and CT air voxels (<-500 HU) if the corresponding sCT voxels were soft tissue (>-200 HU),^2^ see Fig. 1.

**Fig. 2:**
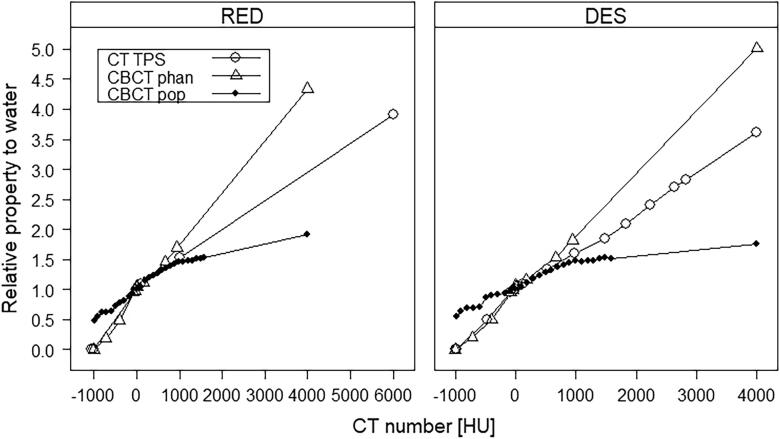
CT calibration curves to relative electron densities (RED, left) and mass densities (DES, right). CT default treatment planning system (TPS, open circles), CBCT phantom curve containing large cylindrical plastic scatter material with known density inserts (CBCT phan, open triangles) and CBCT population curve (CBCT pop, closed circles). CBCT pop curves are shown for patient 8 with a high median absolute error (MeAE) (left) and 1 with a low MeAE (right). The curves were built from patients 1–7 and 9–10, and, 2–10, respectively. Rightmost points are extrapolations except for the TPS CT RED curve.

CT numbers were converted to relative electron densities (RED) and mass densities (DES) using the treatment planning system (TPS) calibration curve for the CT and sCT. For the CBCT RED and DES conversion, a calibration curve based on a CBCT phantom (phan) and a paired CT-CBCT population (pop) of the 9 other patients was applied, as presented in Fig. 2. For the latter, CBCT numbers were averaged in 100 HU bins. The known RED/DES of the corresponding CT bins were then averaged and assigned to build the calibration curve with the paired CBCT bins. Bins with points < 100 were disregarded. CT-CBCT pairs were aligned and corrected similar to the sCT-CBCT pairs prior to building the CBCT pop curves.

The CT numbers, RED and DES of the paired sCT-CT and sCT-CBCT data were averaged in bins of 10 HU or 0.01 RED/DES over all tissues. Bins with points < 100 were again disregarded. The absolute error between the sCT-CT and sCT-CBCT data was calculated for each bin as

(1) AEi=sCTi-CBCTi

where *AE_i_* is absolute error between the mean values of the sCT and CT or CBCT of the *i^th^* bin in HU, RED or DES. The median of the absolute binned errors (MeAE) was then found as

(2) $$\mathrm{MeAE}=\frac{1}{2}\left[{\mathrm{AE}}_{\left\lfloor \left(n+1\right)/2\right\rfloor }+{\mathrm{AE}}_{\left\lceil \left(n+1\right)/2\right\rceil }\right]$$

where *AE_i_* is an ordered list of *n* bins, and ⌊∙⌋ and ⌈∙⌉ are the floor and ceiling function, respectively. Unlike the more commonly reported mean absolute error (MAE) metrics which average the absolute error of all voxels and thus is biased towards a large number of water equivalent voxels [5], [23], the whole CT range of voxels contribute equally from each bin to the MeAE.^3^ The *AE_i_* distributions were subject to a Shapiro-Wilk test and found not to be normally distributed (p < 10^−8^ for all patients). An unpaired Wilcoxon rank-sum test was performed to determine significant difference between the sCT-CT and sCT-CBCT *AE_i_* distributions in HU, RED and DES space. Significance was obtained for p-values <0.05 for the IGRT and sCT quality study.

## 3 Results

The ΔsCT and ΔMRI match differences relative to the CT-CBCT match (set to zero) can be seen in Table 1. The mean difference was ≤2 mm with a maximum standard deviation (std) of 2–4 mm. This was especially pronounced for the CC direction across all patients and CBCTs for individual patients. A similar observation was seen for the pitch rotation, which had a mean around 1° and a std of 1-2°. A few outliers of 6–8 mm and 4-5° were observed for these directions. Significant difference was observed only for the AP directions of all patients. However, this pattern could not be reproduced for the CBCTs of the individual patients. Significance was similar for both ΔMRI and ΔsCT except for the LR direction of patient 9.

**Table 1:** Pooled ΔsCT and ΔMRI match differences for one CBCT of all patients (top), and, all 8 CBCTs of patient 7 (middle) and all 9 CBCTs of patient 9 (bottom). Numbers indicate mean ± 1 standard deviation in mm (translations) and degrees ° (rotations). Significant p-values are in italic font. MRI-CBCT (ΔMRI) and sCT-CBCT (ΔsCT) difference relative to the CT-CBCT registration. AP = anterior-posterior, LR = left–right and CC = cranio-caudal.

*One CBCT of all patients*				
**Direction**	Δ**MRI**	**p-value**	Δ**sCT**	**p-value**
AP [mm]	1.9 ± 1.6	*0.02*	1.5 ± 2.2	*0.04*
CC [mm]	2.0 ± 2.8	>0.1	0.6 ± 4.0	>0.1
LR [mm]	−0.3 ± 1.1	1	−0.5 ± 0.9	>0.1
Pitch [°]	1.1 ± 1.9	>0.1	1.2 ± 1.9	>0.1
Roll [°]	0.0 ± 0.5	1	0.1 ± 0.6	1
Jaw [°]	0.0 ± 0.2	>0.1	−0.2 ± 0.2	>0.09

*All 8 CBCTS of patient 7*				

**Direction**	Δ**MRI**	**p-value**	Δ**sCT**	**p-value**
AP [mm]	−0.1 ± 0.7	>0.1	−0.2 ± 0.1	0.06
CC [mm]	1.9 ± 0.5	*0.04*	0.2 ± 0.2	*0.04*
LR [mm]	−0.2 ± 0.5	>0.1	0.2 ± 0.2	>0.1
Pitch [°]	1.1 ± 0.5	*0.04*	0.8 ± 0.5	*0.04*
Roll [°]	0.1 ± 0.4	>0.1	0.2 ± 0.3	>0.1
Jaw [°]	0.2 ± 0.4	>0.1	0.2 ± 0.3	>0.1

*All 9 CBCTS of patient 9*				

**Direction**	Δ**MRI**	**p-value**	Δ**sCT**	**p-value**
AP [mm]	0.0 ± 0.5	>0.1	0.6 ± 0.3	*0.03*
CC [mm]	1.1 ± 0.4	*0.03*	−0.8 ± 0.3	*0.03*
LR [mm]	0.0 ± 0.4	>0.1	−0.4 ± 0.2	*0.04*
Pitch [°]	−0.2 ± 0.6	>0.1	−0.5 ± 0.6	>0.1
Roll [°]	−0.4 ± 0.3	*0.03*	−0.7 ± 0.3	*0.03*
Jaw [°]	−0.5 ± 0.3	*0.03*	−0.4 ± 0.4	*0.04*

The MeAE of all patients are shown in Table 2. Overall, CBCT HU or phan-based RED/DES difference did not provide a similar estimate for the sCT quality as compared to the true CT-sCT difference. A CBCT pop-based RED/DES difference, however, provided this estimate in most cases, i.e. non-significance from the CT-sCT difference.

**Table 2:** The median absolute error for CT numbers in Hounsfield units (MeAE_HU_) of sCT-CT (CT) and sCT-CBCT (CBCT, left), relative electron densities (MeAE_RED_, middle) and relative mass densities (MeAE_DES_, right). The CBCT RED/DES conversion is either made with a phantom (CBCT_phan_) or population (CBCT_pop_) based calibration curve (see Fig. 2). Last row indicates mean (M) and standard deviation (SD) for all patients. RED/DES MeAE values are multiplied by 10^3^.

	MeAE_HU_			MeAE_RED_∙10^3^					MeAE_DES_∙10^3^				
Patient	CT	CBCT	p-value	CT	CBCT_phan_	p-value	CBCT_pop_	p-value	CT	CBCT_phan_	p-value	CBCT_pop_	p-value
1	82	208	≪0.05	32	236	≪0.05	42	>0.1	44	267	≪0.05	43	>0.1
2	67	169	≪0.05	18	200	≪0.05	42	>0.1	22	232	≪0.05	38	>0.1
3	161	253	≪0.05	81	193	≪0.05	41	>0.1	112	278	≪0.05	57	>0.1
4	77	313	≪0.05	38	341	≪0.05	51	>0.1	48	383	≪0.05	61	>0.1
5	123	159	0.078	31	186	≪0.05	47	>0.1	33	213	≪0.05	39	>0.1
6	87	411	≪0.05	51	367	≪0.05	87	≪0.05	65	434	≪0.05	113	≪0.05
7	134	336	≪0.05	67	326	≪0.05	92	>0.1	91	377	≪0.05	115	>0.1
8	156	440	≪0.05	81	391	≪0.05	134	≪0.05	93	467	≪0.05	165	≪0.05
9	126	271	≪0.05	77	278	≪0.05	71	>0.1	95	320	≪0.05	99	>0.1
10	78	222	≪0.05	57	224	≪0.05	54	>0.1	61	262	≪0.05	61	>0.1
M ± SD	109 ± 35	278 ± 96		53 ± 23	274 ± 77		66 ± 30		66 ± 30	323 ± 87		79 ± 42	

## 4 Discussion

sCT generation methods have demonstrated <0.5–1% agreement with CT-based dose calculations and it is thus questionable if further advancements in sCT generation are clinically meaningful. Hence more attention should be given to other steps in the RT chain and here, we assessed the agreement in MRI-only IGRT and sCT quality verification for the pelvis.

Overall, the average deviations between CT and MRI-only based IGRT seem acceptable whether the MRI or sCT is used as reference. However, significant differences were observed which depended on patient cohort (Table 1, top) or the CBCT course of individual patients (Table 1, middle and bottom) and therefore no unambiguous conclusions can be drawn. Caution is therefore advised in making general IGRT statements based on patient cohorts’ single CBCT. The magnitude in differences and outliers were especially pronounced for the CC and pitch directions whereas deviations in the other directions were around 1 mm and 1° or less in line with previous studies [14], [15]. It is likely that this is caused by the relatively short longitudinal (long) MRI FOV of 64–96 mm. This could result in incorrect combinations of CC and pitch that lead to a (favored) reduction in the registration cost function that is similar to a correct one. A MRI long FOV > 100 mm is therefore suggested at the expense of increased MRI scanning time.

To reduce the influence of differences between the sCT, CT and CBCT scans not caused by the sCT generation method, we 1) aligned the anatomy through ridged registration, 2) filled inconsistent air cavities with water and 3) adjusted for HU intensity by transforming tissue voxels into (electron) densities. This seems like a clinically feasible approach given the data available although not ideal. Deformable registration is another approach to minimize these differences but introduce additional challenges for verifying the correctness of the deformation field [24]. The MeAE metric suggests an error estimate of the sCT quality similar to a CT reference if the CBCT voxel values are transferred to RED or DES space using a population (pop) based calibration curve. This curve behaves quite differently as compared to the TPS and traditional phantom based calibration curves (Fig. 2). A major contribution to the CBCT HU numbers is scattered radiation [25], [26], [27]. In the pop curves, the true patient scattering geometries result in more photons being scattered away from detectors when crossing low density region and into the detectors in high density regions, resulting in a more even curve over the CT range. Given the ever-developing reconstruction algorithms and equipment, the pop curves are likely to be dependent on vendor, model version and anatomical site, see e.g. our previous pop curve for the brain [16].

**Fig. 3:**
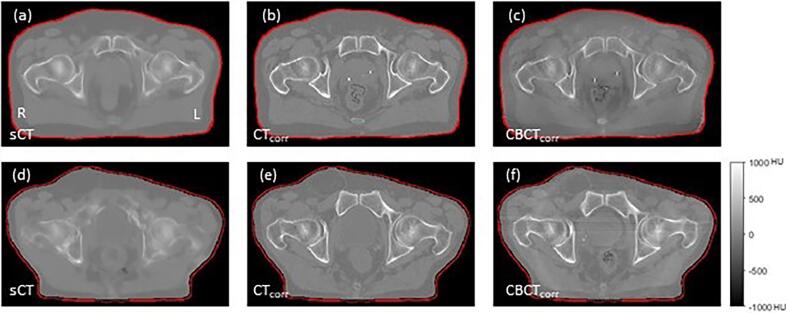
Illustration of synthetic CT image quality for a low (patient 1, panels a–c) and high (patient 8, panels d-f) median absolute error (MeAE). (a) and (d) synthetic CT = sCT. Patient orientation indicated by right = R and left = L. (b) and (e) corrected CT = CT_corr_ with air cavities and body outline difference as compared to the sCT filled with water. (c) and (f) corrected CBCT = CBCT_corr_. The red contour is the body outline of the sCT. Grayscale is in Hounsfield units (HU). CT, sCT and CBCT voxel resolution and re-sampling are identical to Fig. 1.Corresponding population based (pop) CBCT calibration curves for these patients can be found in Fig. 2. (For interpretation of the references to colour in this figure legend, the reader is referred to the web version of this article.)

The MeAE shows a low and high DES value of 0.04 and 0.17 for patient 1 and 8, respectively. By inspection of Fig. 3, it is clear that the bony anatomy of the sCT is much better predicted in patient 1 than patient 8. This suggests that the MeAE metric could help flag a sCT of unacceptable quality for clinical use. A sCT-CBCT MeAE value above 0.1 DES or RED could act as an initial action level for required inspection. The corresponding MAE DES values were 0.042 and 0.048 for patient 1 and 8, respectively, leaving little room for discrimination in image quality using this metric.

In conclusion, both the MRI and sCT can be used for MRI-only CBCT-based IGRT in the pelvis but caution is advised for longitudinal FOVs < 100 mm. The CBCT seems adequate to assess pelvic sCT quality if converted to RED or DES using a population-based calibration. A MeAE of 0.1 DES is suggested as a potential action level for inspection of sCT quality.

## Declaration of Competing Interest

The authors declare that they have no known competing financial interests or personal relationships that could have appeared to influence the work reported in this paper.

## Footnotes

1 For patient 9, a 2nd CBCT was acquired on the 3rd fraction due to a large rectal air pocket observed at 1st fraction. The 2nd CBCT was subsequently used for sCT quality assessment.

2 Most sCT methods overwrite air pockets in the input MR with a water equivalent HU.

3 The sCT predictions often agree well with the real CT numbers in the region around 0 HU while a lower agreement typically is found in the bone area. The median value is further less sensitive to outliers as compared to the mean value.
